# Supplementary material for: Place-based approaches to improve health and development outcomes in young children: A scoping review
Source: PLoS One. 2021 Dec 23;16(12):e0261643. doi: 10.1371/journal.pone.0261643 (PMC8700019; doi:10.1371/journal.pone.0261643)
Supplement: S1 Appendix — (DOCX) [file pone.0261643.s001.docx]

**S1 Appendix. Example search strategy – EMBASE**

| 1. (intervention* or initiative* or program* or trial).mp. [mp=title, abstract, heading word, drug trade name, original title, device manufacturer, drug manufacturer, device trade name, keyword] |  |
| --- | --- |
| 2. ("place based" or "place-based" or "area based" or "area-based" or "location based" or "location-based" or "neighbourhood based" or "neighbourhood-based" or "neighborhood based" or "neighborhood-based" or "community based" or "community-based" or "area level" or "area-level" or "location level" or "location-level" or "neighbourhood level" or "neighbourhood-level" or "neighborhood level" or "neighborhood-level" or "community level" or "community-level" or "complex community" or "collective impact").mp. [mp=title, abstract, heading word, drug trade name, original title, device manufacturer, drug manufacturer, device trade name, keyword] |  |
| 3. (infant* or child* or parent* or family or families).mp. [mp=title, abstract, heading word, drug trade name, original title, device manufacturer, drug manufacturer, device trade name, keyword] |  |
| 4. (evaluat* or outcome* or impact* or effect* or efficacy or pilot or feasib* or protocol).mp. [mp=title, abstract, heading word, drug trade name, original title, device manufacturer, drug manufacturer, device trade name, keyword] |  |
| 5. (socioeconomic or "socio-economic" or "socio economic" or SES or SEP or poverty or disadvantage* or vulnerable or inequality or inequalities or inequity or inequities or wellbeing or "well-being").mp. [mp=title, abstract, heading word, drug trade name, original title, device manufacturer, drug manufacturer, device trade name, keyword] |  |
| 6. 1 and 2 and 3 and 4 and 5 |  |
| 7. 6 not (bangladesh or india* or HIV or AIDS or kenya* or uganda* or ghana or ethiopia* or pakistan or iran* or korea* or nigeria* or "oral health" or Sudan or Rwanda or autism or Vietnam or drug* or "Sri Lanka" or Nepal or Indonesia or dementia or disabilit* or "brain injury").mp. [mp=title, abstract, heading word, drug trade name, original title, device manufacturer, drug manufacturer, device trade name, keyword] |  |
| 8. 2 and 3 and 4 and 5 |  |
| 9. 8 not (bangladesh or india* or HIV or AIDS or kenya* or uganda* or ghana or ethiopia* or pakistan or iran* or korea* or nigeria* or "oral health" or Sudan or Rwanda or autism or Vietnam or "Sri Lanka" or Nepal or Indonesia or dementia or disabilit* or "brain injury").mp. [mp=title, abstract, heading word, drug trade name, original title, device manufacturer, drug manufacturer, device trade name, keyword] |  |
