# Supplementary material for: Place-based approaches to improve health and development outcomes in young children: A scoping review
Source: PLoS One. 2021 Dec 23;16(12):e0261643. doi: 10.1371/journal.pone.0261643 (PMC8700019; doi:10.1371/journal.pone.0261643)
Supplement: S2 Appendix — (DOCX) [file pone.0261643.s002.docx]

**S2 Appendix. Quality of impact study based on fit-for-purpose**

|  | **CfC** | **ABC Programme** | **Sure Start** | **NNI** | **Flying Start** | **Best Start** | **Starting Well** | **GFC** | **TFD** | **First Steps** | **Smart Start** | **NEYAI** |
| --- | --- | --- | --- | --- | --- | --- | --- | --- | --- | --- | --- | --- |
| **Broad range of outcome measures?** | **Yes** | **Yes** | **Yes** | **Some** | **Yes** | **Yes** | **Some** | **No** | **Some** | **Some** | **No** | **Yes** |
| **Measures are a good match for the desired outcomes for the program?** | **✓** | **✓** | **✓** | **✓** | **✓** | **Some** | **Some** | **Some** | **✓** | **Some** | **Some** | **✓** |
| **Evaluation designed before or at time of implementation?** | **✓** | **x** | **X** | **unclear** | **X** | **✓** | **X** | **X** | **X** | **X** | **X** | **X** |
| **Evaluation allowed time for full implementation?** | **✓** | **✓** | **✓** | **✓** | **✓*** | **X** | **X** | **X** | **X** | **✓** | **✓** | **X** |
| **Multiple impact points measured?** | **✓** | **x** | **✓** | **X** | **✓*** | **X** | **✓** | **✓** | **X** | **✓** | **✓** | **X** |
| **Measures change at the population level?** | **✓** | **x** | **✓** | **✓** | **✓** | **✓** | **X** | **X** | **✓X**** | **X** | **✓** | **X** |
| **Appropriateness of comparison group?** | **✓** | **n/a** | **Partly***** | **✓** | **✓** | **✓** | **✓** | **✓** | **✓** | **✓** | **✓** | **X** |
| **Score** | **7** | **3** | **5** | **4.5** | **6** | **4.5** | **3** | **5** | **3.5** | **4** | **4.5** | **2** |
| **Rating** | **high** | **low** | **medium** | **medium** | **high** | **medium** | **low** | **medium** | **low** | **medium** | **medium** | **low** |

* The main impact evaluation was conducted 1 year after the initiative was considered to be fully implemented and only collected one wave of impact data. However subsequent evaluations using routinely collected data were undertaken when the initiative was more mature.

** Change was measured at the school level

*** Comparison group was changed due to rapid upscaling of Sure Start. Millennium Cohort Study was subsequently used as comparison group and this group was relatively more advantaged, timing of measurements differed by up to two years.

**HIGH: 6-7 MEDIUM: 4-5 LOW: 0-3**
