# Supplementary material for: Place-based approaches to improve health and development outcomes in young children: A scoping review
Source: PLoS One. 2021 Dec 23;16(12):e0261643. doi: 10.1371/journal.pone.0261643 (PMC8700019; doi:10.1371/journal.pone.0261643)
Supplement: S3 Appendix — (DOCX) [file pone.0261643.s003.docx]

**S3 Appendix. Tables of study reported outcomes by categories and domains**

**S3 Table 1. Pregnancy and birth domains**

| Initiative | A1 Birthweight & age | A2 Pregnancy/ delivery | A3 Prenatal & infant health | A4 Breastfeeding | **Pregnancy & Birth Summary** |
| --- | --- | --- | --- | --- | --- |
| **Comparison group** | | | | | |
| CfC (AUS) | - | - | - | - | **None measured** |
| Sure Start (UK)^1^ | No/weak | - | - | No/weak | **2 no/weak** |
| NNI (UK) | - | - | - | - | **None measured** |
| Flying Start (Wales) | +ve^2^ | +ve^2^ | No/weak | No/weak | **2 +ve**  **2 no/weak** |
| Best Start (AUS) | - | - | No/weak | +ve | **1 +ve**  **1 no/weak** |
| First Steps (USA) | - | - | - | - | **None measured** |
| Smart Start (USA) | - | - | - | - | **None measured** |
| GFC (USA) | +ve, y | - | - | - | **1 +ve, sustained** |
| TFD (CAN) | - | - | - | - | **None measured** |
| NEYAI (Ireland) | - | - | - | - | **None measured** |
| Starting Well (Scotland) | - | - | - | - | **None measured** |
| **Summary** |  |  |  |  | **9 measured**  **5 +ve, 1 sustained**  **4 no/weak** |
| **No comparison group** | | | | | |
| ABC (Ireland) | - | - | - | - | **None measured** |

NOTE: +ve indicates positive or –ve indicates negative effect at P<=0.05; If measured more than once, sustained effect indicated yes/no (y/n)

1 Due to the change in impact study design of Sure Start, not all measures were repeated. Items that were only measured once post-baseline are: A1 birthweight and age and A4 breastfeeding,

2 Data sourced from administrative data demonstration project reported with noted methodological limitations

**S3 Table 2. Child domains**

| Initiative | B1 Physical health | B2 Emotional and behavioural functioning | B3 Temperament/ Self-regulation | B4 Attendance at formal childcare/ early learning | B5 Developmental Status | B6 School readiness | B7 Educational Attainment & Attendance | B8 Language/ cognition | B9 Child safety | **Child Summary** |
| --- | --- | --- | --- | --- | --- | --- | --- | --- | --- | --- |
| **Comparison Group** | | | | | | | | | | |
| CfC (Aus) | -ve, n | no/weak | - | - | - | - | no/weak | - | **-** | **2 no/weak**  **1 –ve, not sustained** |
| Sure Start (UK)^1^ | +ve, y^2^ | +ve | +ve | - | - | - | No/weak | No/weak | **-** | **3 +ve, 1 sustained**  **2 no/weak** |
| NNI (UK) | - | - | - | No/weak^3^ | - | - | - | - | **-** | **1 no/weak** |
| Flying Start (Wales) | +ve | No/weak | No/weak | - | +ve | - | +ve | No/weak | **-** | **3 +ve**  **3 no/weak** |
| Best Start (AUS) | No/weak | - | - | No/weak | - | - | No/weak | No/weak | **-** | **4 no/weak** |
| First Steps (USA) | - | - | - | - | Mixed | Mixed^4^ | Mixed^4^ | - | **-** | **3 mixed** |
| Smart Start (USA) | - | - | - | - | - | - | +ve, y | - | **-** | **1 +ve, sustained** |
| GFC (USA) | - | - | - | - | - | - | - | - | **-** | **None measured** |
| TFD (CAN) | No/weak | +ve | No/weak | - | - | - | - | No/weak | **-** | **1 +ve**  **3 no/weak** |
| NEYAI (Ireland) | No/weak | No/weak | No/weak | - | - | - | - | No/weak | **-** | **4 no/weak** |
| Starting Well (Scotland) | - | - | - | - | - | - | - | - | **-** | **None measured** |
| **Summary** |  |  |  |  |  |  |  |  |  | **31 measured**  **8 +ve, 2 sustained**  **19 no/weak**  **1 –ve, not sustained**  **3 mixed** |
| **No comparison group** | | | | | | | | | | |
| ABC (Ireland) | - | +ve | - | - | - | +ve |  | +ve | **-** | **3 +ve** |

NOTE: +ve indicates positive or –ve indicates negative effect at P<=0.05; If measured more than once, sustained effect indicated yes/no (y/n)

1 Due to the change in impact study design of Sure Start, not all measures were repeated. Items that were only measured once post-baseline are: B2 emotional and behavioural functioning, and B3 temperament/self-regulation

2 Better physical health at 3 and at 5 but in different measures

3 Data based on population sample and targeted population sample, not intervention sample (ie users of the service)

4 Greater positive effects in later years of the initiative

**S3 Table 3. Parent domains**

| Initiative | C1 Physical health status | C2 Mental health status | C3 Health risk behaviours | C4 Social support (personal) | C5 Employment Status/ Movement off Benefits | **Parent Summary** |
| --- | --- | --- | --- | --- | --- | --- |
| **Comparison Group** | | | | | | |
| CfC (Aus) | No/weak | -ve, y | - | - | +ve, n | **1 +ve, not sustained**  **1 no/weak**  **1 –ve, sustained** |
| Sure Start (UK) | No/weak | -ve, n | No/weak | +ve, y^1^ | +ve, n | **2 +ve, 1 sustained**  **2 no/weak**  **1 –ve, not sustained** |
| NNI (UK) | - | - | - | - | +ve | **1 +ve** |
| Flying Start (Wales) | - | No/weak | - | No/weak | - | **2 no/weak** |
| Best Start (AUS) | - | - | - | - | - | **None measured** |
| First Steps (USA) | - | - | - | - | - | **None measured** |
| Smart Start (USA) | - | - | - | - | - | **None measured** |
| GfC (USA) | - | - | - | - | - | **None measured** |
| TFD (CAN) | - | - | - | - | - | **None measured** |
| NEYAI (Ireland) | - | - | - | - | - | **None measured** |
| Starting Well (Scotland) | - | +ve, n | - | - | - | **1 +ve, not sustained** |
| **Summary** |  |  |  |  |  | **12 measured**  **5 +ve, 1 sustained**  **2 –ve, 1 sustained**  **5 no/weak** |
| **No comparison group** | | | | | | |
| ABC (Ireland) | - | - | - | - | - | **None measured** |

NOTE: +ve indicates positive or –ve indicates negative effect at P<=0.05; If measured more than once, sustained effect indicated yes/no (y/n)

1 Maternal life satisfaction

**S3 Table 4. Family domains**

| Initiative | D1 Parenting style/ confidence | D2 Partner relationship | D3 Reading with child | D4 Activities with child | D5 Other family functioning | D6 Household safety | **Family Summary** |
| --- | --- | --- | --- | --- | --- | --- | --- |
| **Comparison Group** | | | | | | | |
| CfC (Aus) | +ve, y | no/weak | +ve | no/weak | - | No/weak | **2 +ve, 1 sustained**  **3 no/weak** |
| Sure Start (UK) | +ve, y | No/weak^1^ | +ve, n | - | +ve, y | - | **3 +ve, 2 sustained**  **1 no/weak** |
| NNI (UK) | - | - | - | - | - | - | **None measured** |
| Flying Start (Wales) | No/weak | - | No/weak | No/weak | - | No/weak | **4 no/weak** |
| Best Start (AUS) | No/weak | - | - | - | - | - | **1 no/weak** |
| First Steps (USA) |  |  |  | - | - | - | **None measured** |
| Smart Start (USA) | - | - | - | - | - | - | **None measured** |
| GFC (USA) | - | - | - | - | - | - | **None measured** |
| TFD (CAN) | - | - | - | - | - | - | **None measured** |
| NEYAI (Ireland) | - | - | - | - | - | - | **None measured** |
| Starting Well (Scotland) | - | - | - | - | No/weak | - | **1 no/weak** |
| **Summary** |  |  |  |  |  |  | **15 measured**  **5 +ve, 3 sustained**  **10 no/weak** |
| **No comparison group** | | | | | | | |
| **ABC (Ireland)** | +ve | - | - | +ve | - | - | **2 +ve** |

NOTE: +ve indicates positive or –ve indicates negative effect at P<=0.05; If measured more than once, sustained effect indicated yes/no (y/n)

1 Partner involvement

**S3 Table 5. School & community domains**

| Initiative | E1 Community involvement (eg volunteering, coaching) | E2 Social cohesion/ belonging | E3 Neighbourhood safety | E4 Service use (incl health, development, family support, schools) | E5 Service quality (incl health, development, family support, childcare, early learning & schools) | E6 Service availability/ access | E7 Child friendly community | **School & Community Summary** |
| --- | --- | --- | --- | --- | --- | --- | --- | --- |
| **Comparison Group** | | | | | | | | |
| CfC (AUS) | +ve, y | No/weak | No/weak | - | - | No/weak | **-** | **1 +ve, sustained**  **3 no/weak** |
| Sure Start (UK) | -ve, n | - | No/weak | +ve, n | - | - | **-** | **1 +ve, not sustained**  **1 –ve, not sustained**  **1 no/weak** |
| NNI (UK) | - | - | - | - | - | - | **-** | **None measured** |
| Flying Start | - | - | - | +ve | +ve | +ve | +ve | **4 +ve** |
| Best Start (AUS) | - | - | - | +ve | - | No/weak | +ve | **2 +ve**  **1 no/weak** |
| First Steps (USA) | - | - | - | - | - | - | **-** | **None measured** |
| Smart Start (USA) | - | - | - | - | - | - | **-** | **None measured** |
| GFC (USA) | - | - | - | - | - | - | **-** | **None measured** |
| TFD (CAN) | - | - | - | - | - | - | **-** | **None measured** |
| NEYAI (Ireland) | - | - | - | - | - | - | **-** | **None measured** |
| Starting Well (Scotland) | - | - | - | +ve, y | +ve, y | - | **-** | **2 +ve, sustained** |
| **Summary** |  |  |  |  |  |  |  | **16 measured**  **10 +ve, 3 sustained**  **1 –ve**  **5 no/weak** |
| **No Comparison Group** | | | | | | | | |
| ABC (Ireland) | - | - | - | - | - | - | **-** | **None measured** |

NOTE: +ve indicates positive or –ve indicates negative effect at P<=0.05; If measured more than once, sustained effect indicated yes/no (y/n)
