# Supplementary material for: Place-based approaches to improve health and development outcomes in young children: A scoping review
Source: PLoS One. 2021 Dec 23;16(12):e0261643. doi: 10.1371/journal.pone.0261643 (PMC8700019; doi:10.1371/journal.pone.0261643)
Supplement: Attachment — Submitted filename: Response to Reviewers.docx [file pone.0261643.s005.docx]

**Place-based approaches to improve health and development outcomes in young children: a scoping review [PONE-D-20-30217]**

**Response to editor and reviewer comments**

| Number | Editor/Reviewer Comments | Response to Editor/Reviewer |
| --- | --- | --- |
| 1. | 1) Please ensure that your manuscript meets PLOS ONE's style requirements, including those for file naming. The PLOS ONE style templates can be found at  [https://journals.plos.org/plosone/s/file?id=wjVg/PLOSOne_formatting_sample_main_body.pdf](https://protect-au.mimecast.com/s/N6VLCZYM5Vu2wgD4uyroX3?domain=journals.plos.org) and  [https://journals.plos.org/plosone/s/file?id=ba62/PLOSOne_formatting_sample_title_authors_affiliations.pdf](about:blank) | Thank you, we believe we have now updated the manuscript to comply with all PLOS ONE’s style requirements. Please advise if further changes are required. |
| 2. | 2) Please include captions for your Supporting Information files at the end of your manuscript, and update any in-text citations to match accordingly. Please see our Supporting Information guidelines for more information: [http://journals.plos.org/plosone/s/supporting-information](about:blank). | We have now provided these captions at the end of our manuscript and updated in-text citations to match accordingly. |
| 3. | Reviewer #1: This Systematic Review has been carefully conducted with strict adherence to the PRISMA principles. The conclusions reached are plausible. It also clearly brings out the varied problems with studies evaluating the place-based approaches to improve health and development outcomes in developed countries | Thank you |
| 4. | Reviewer #2: An interesting investigation based on much time and effort. Please excuse any repetition below. | Thank you for this comment and for the detailed feedback provided. We appreciate the time and effort taken to review the paper. We have addressed all comments (see our responses below) and believe it has significantly strengthened the paper.  Lines and page numbers referenced below are for the untracked revised paper. |
| 5. | My overall assessment is that this is a scoping vs systematic review; | We have amended the paper and re-framed it as a Scoping Review. See also response to point 9 below |
| 6. | that the inclusion criteria--including population and outcomes, need to be more fully and clearly stated such that the results reported are in better alignment. | We have reframed the inclusion criteria to align with this suggestion. For more information see responses to the more detail feedback provided below. |
| 7. | Tables and the text should be better referenced, e.g. Tables do not have references. Given the (very good and much appreciated) approach of collecting multiple reports of single studies/initiatives, a table showing references grouped by initiative would be useful. Take a look at an included studies table in a Cochrane Systematic review to see an example if my suggestion is not clear. | We have now included an additional column in Tables 3, 4 & 5 that contains the relevant references (author, year) for initiatives, and included the PLOS ONE style references. |
| 8. | Based on above excerpts, one from the abstract, one from the text, I am not sure the aim of the review is stated consistently. | The excerpts were not included in the feedback provided to the authorship team, however we have amended the wording of the aim of the review to be more consistent across the abstract (Lines 24-28) and the manuscript (Lines 120-125). |
| 9. | RE type of review: I can see that a number of standard systematic review processes were followed, e.g. documented literature search; quality assessment, but these procedures are also part of scoping review methodology. The inclusion of any study design supports, I think, the idea that this is a scoping review, as does the use of single (unvalidated?) quality assessment checklist across multiple designs (line 192). There are, as you know, standard and validated Risk of Bias and quality assessment tools for a variety of study designs and use of such tools is part of systematic review methodology. I notice the authors reference "A schema for evaluating evidence of public health interventions' and this source provides guidance on assessing studies based on design. In this review, studies with similar designs do not appear to be grouped together and analysed--which may have been possible since I am uncertain as to whether heterogeneity referred to all studies, regardless of design or whether it was found within studies using the same design. Further, in systematic reviews, quality is less the issue than risk of bias inherent in various study designs. Issues of quality are typically dealt with by using GRADE in conjunction with RoB in systematic reviews in order to provide an estimation of the strength of evidence by outcome and across studies.  This article about scoping reviews references a number of the earlier/foundational papers on the method: [https://www.journalofhospitalmedicine.com/jhospmed/article/202729/hospital-medicine/methods-research-evidence-synthesis-scoping-review](https://protect-au.mimecast.com/s/Eyj5C2xZ6vCO43RlFM2B3n?domain=journalofhospitalmedicine.com) and may provide an indication of my opinion regarding my assessment that this is a scoping vs systematic review. | We have been guided by the reviewer’s expertise in this matter and have amended the review as a Scoping Review, including the PRISMA reporting and referencing the JBI Scoping Review guidelines. |
| 10. | I believe it would be useful to better delineate inclusion criteria following, roughly PICOS-ish categories; a clear delineation of outcomes or outcome data of interest would, for instance be quite useful and would better prepare the reader for the results sections. | We have amended the inclusion criteria section to comply with this suggestion. For further detail please see response to point 21 below. |
| 11. | I believe some of the information that seems either under-explained or missing from the text of the review is found in Tables, but I would like the Tables to support the text vs having to scrutinize the tables to better understand the narrative description of findings. | We have included more explanatory text throughout the document, particularly in the sections specified in the more detailed feedback below. |
| 12. | A number of detailed remarks below. |  |
| 13. | Table 2: I'm not sure i understand the numbers reported for Peer reviewed only; Grey only; and Mixed. | We have amended this heading to be clear that we are reporting these findings by Initiative. |
| 14. | LIne 193-194: I'd be interested to see references here to support the statement re "standard evaluation methods..." | We have amended the wording of this statement slightly added references to support it (Lines 245-247). |
| 15. | Line 380; I believe references should be provided here so we know which 12 studies are being discussed; same comment for lines 385-386, 402. etc. | We have now included additional referencing throughout the manuscript, wherever a number of initiatives or specific initiatives are referred to. |
| 16. | Table 4: I believe there should be a column for design vs relying on Comparison group and no comparison group sub-sections. I'd also like to see references in this and other tables. | We have added two columns to Table 4: one for references and one for the outcome study design for each initiative. |
| 17. | PRISMA is referred to as the guide for the review, but PRISMA tells authors what to report, not how to "do" or conduct a systematic review; referencing PRISMA in this way is common across the literature, but it is incorrect. The Cochrane Handbook is an example of a source for methodological guidance and I believe it is cited in the reference list of this review; if it was the guide of the authors' methods, I'd suggest stating this in the text. Having said this, referencing methodological guidance on scoping reviews may be more appropriate. | We have referred to the JBI methodological guidance for Scoping Reviews, noting that while we did not use this guidance at the time of writing the review, we have cross checked our review against these guidelines to ensure broad consistency.  We have also now amended the review to report against the PRISMA guidelines for Scoping Reviews.  (Lines 127-129) |
| 18. | Search and Search Methods: Quite well done; especially in the choice of multiple databases. However, I believe it would have been worthwhile to explore controlled vocabulary in at least Embase and Medline/Pubmed where there are thesauri. Regarding databases: ProQuest and Informit are platforms and/or database providers. Both ProQuest and Informit provide access to many databases. I notice that Informit, for instance, offers the Australian Education Index and Australian Public Affairs Information Service, to name two. Were searches in ProQuest and Informit simply done across multiple databases? If so, make this clear, and perhaps name a few of the most relevant (to this review) databases from each database provider; if, on the other hand, only specific ProQuest and Informati databases were searched, these should be named. | We note the preference to explore controlled vocabulary in Embase and Medline and will explore these options in future systematic reviews.  We have now updated the text to clarify that we searched the ProQuest Central database and all databases in Informit (Lines 134-135). |
| 19. | Terminology: I'd suggest referring to the current work as a "review" vs study; use the word study to describe the included studies. For reference, there is a section, pg 4, called "The Current Study." | Thank you we have now updated all references to our ‘study’ in the text to ‘review’ (eg lines 48, 88, etc). |
| 20. | Peer reviewed. I am not sure this phrase or criteria should be used--especially since grey literature reports--which are often not peer reviewed--were sought during the search. | We are unsure of the context in which this comment is made.  We have removed the references to peer reviewed from the section ‘The current study’.  It is accurate to state that we sought both peer reviewed and grey literature reports during the search, and have outlined the different search processes we used to capture both types of literature. We nevertheless wanted to distinguish between different source materials we found and relied upon for our review. |
| 21. | In Eligibility Criteria, one subheading title is 'Initiative selection". It might be a bit clearer to either omit this subtitle, or rename it inclusion criteria. It might also be useful to use bullets or brief, descriptive phrases prior to the description of each criteria, e.g. Item 1: Population: Socially disadvantaged children (infancy to 4 years) and pregnant women. Item 2: Location: high-income countries Sponsoring organization (or something like that): governmentally administered program. The question of the level of government seems more an outcome. Number of sites/extent of program: more than one site Item 4 seems to repeat information in item 3 where level of government is mentioned. Item 5: Reported program evaluation examining more than one child-focussed outcome. | Thank you, we have reworked the Eligibility criteria section (p7-9) to comply with these recommendations, Scoping Review guidelines and Cochrane style guidelines for inclusion/exclusion criteria |
| 22. | Lines 153 and 154 are a bit unclear; see above suggestion to clarify inclusion criteria, eg "more than one...outcome" I think a subheading for this paragraph, Exclusion Criteria, would be useful. | See point 21. above |
| 23. | Line 159: Article Selection Here I would describe, using standard systematic review language, the Screening Process. E.g presumably title/abstracts were screened first with eligible or potentially eligible studies moved forward for full-text review. Was screening undertaken independently by two authors? I suggest clearly stating the process. | Now described (Lines 183-185) |
| 24. | Lines 160 Re. the distinction between Primary and Secondary "articles" suggests you have taken a "study based" approach, similar to that used by Cochrane reviews where multiple references for a given study are collated and examined for data. This is a very good idea and demonstrates your thoroughness. | Thank you |
| 25. | Line 163 refers to "additional articles"; use the word "study" or "study reports"; I'd also suggest sticking with the primary/secondary terminology. | We have now updated this section to use primary/secondary terminology |
| 26. | I would like to see a section title Outcomes where the outcomes are detailed. On Page 18, summaries of the Outcomes or Data of interest begin, e.g. Size and selection of delivery areas; theories of change... but I'd like to see these delineated earlier. Table 1 mentions outcome domains, but these do not seem to correspond to the narrative summary beginning on pg 18. | We’ve now added a section ‘Types of outcome measures and other data items of Interest’ p9-10. We collect both ‘outcome data’ to determine whether place-based initiatives are effective, as well as a range of other data items of interest to answer our other research items (methods and methodologies for evaluating place-based initiatives, and the context in which place-based initiatives are implemented and evaluated) so we have tried to differentiate the two to avoid confusion. |
| 27. | LIne 171: "articles were excluded if..." This is an eligibility criteria; I suggest adding this information to the Eligibility Section by creating a sub-heading(s) for "publication type" and "study design" where you would state, positively, the designs you considered eligible and ineligible; it would be worthwhile to describe clearly acceptable designs, e.g. pilot studies; pre-post, cohort, controlled, etc. Line 171 also suggests you included "reviews"--I would like to see this defined because a "review" does not sound like a "study." Be specific in the Inclusion/Eligibility section vs later stating what you excluded. | We now have sub-headings of ‘Types of studies’, ‘Types of place-based initiatives’ and ‘Selection of sources of evidence’ (p7-8) which we hope now clearly states positively what we included and excluded and which complies with the relevant guidelines for conducting a Scoping Review. |
| 28. | Line 172: here you are restating what should be clear from the Eligibility section, eg. of course you are excluding programs that are not place-based initiatives. | This sentence referred to some papers we found which described place-based papers but contained no evaluative data however we have now removed it. |
| 29. | Line 173: what is a "local evaluation"? | Local evaluation is described briefly in the Introduction (Line 83-84), and in line with your suggestion in point 33 below we have broken down the Evaluation Design section into sub-headings and provided a brief definition at the beginning of each paragraph. |
| 30. | Line 173-174: duplication of information in a higher quality source". I do not understand this and, systematic reviews do not exclude studies based on "quality" or high risk of bias; instead, these weaknesses are taken into account in statistical or narrative synthesis. | We have reworded this sentence to clarify that we did not exclude any studies, but rather used higher quality evidence when determining which source to use when the same outcome data was reported in both grey and peer reviewed literature. |
| 31. | Line 174-175: All publications...this is a clear statement, but should be positioned earlier in the paragraph when describing how you grouped/collated multiple reports of the same study. | Thank you we have positioned this statement earlier in the paragraph (Lines 185-186). |
| 32. | Table 1, Line 190: I'm not sure what the "ratings" in the title of this table refer to. | ‘Ratings’ referred to the quality ratings however we have now removed this so that the column is titled ‘Data items and categories’ |
| 33. | line 310: Evaluation Designs. I believe this section tries to break out and describe different approaches to evaluation methods or models used in the included studies, have I got that right? If so, then I would say the language and headings in this section need reworking. Based on line 317 it sounds like program evaluations may be multi-faceted and include various approaches or elements such as impact evaluation, process evaluation, local evaluations, and cost analyses. Earlier in the paper I do not see the phrases referring to these elements. I also think these elements should be briefly defined, especially if subsequent headings refer to them. I notice in a note to Table 3 the terms impact/process etc are mentioned, but explication in the text of the review would be helpful. | Thank you we have now provided further explanatory text in this section (pages 24-29) , and included additional sub-headings and definitions for the different elements of evaluation. |
| 34. | Line 379 "impact (or outcome) evaluation studies varied considerably in design". Does this mean that "impact studies" are a type of study, or is "impact evaluation" a characteristic of program evaluation? | We hope that the additional explanatory text that has been provided in this section has clarified this. |
