# Supplementary material for: Place-based approaches to improve health and development outcomes in young children: A scoping review
Source: PLoS One. 2021 Dec 23;16(12):e0261643. doi: 10.1371/journal.pone.0261643 (PMC8700019; doi:10.1371/journal.pone.0261643)
Supplement: Attachment — Submitted filename: Response to Reviewers October 21.docx [file pone.0261643.s006.docx]

**Place-based approaches to improve health and development outcomes in young children: a scoping review [PONE-D-20-30217]**

**Response to editor and reviewer comments**

| Number | Editor/Reviewer Comments | Response to Editor/Reviewer |
| --- | --- | --- |
| 1. | Journal Requirements:  Please review your reference list to ensure that it is complete and correct. If you have cited papers that have been retracted, please include the rationale for doing so in the manuscript text, or remove these references and replace them with relevant current references. Any changes to the reference list should be mentioned in the rebuttal letter that accompanies your revised manuscript. If you need to cite a retracted article, indicate the article’s retracted status in the References list and also include a citation and full reference for the retraction notice. | We have carefully reviewed our reference list and are confident all references are complete and correct. There are no changes to our references other than formatting and links. |
| 2. | Editor comments:  Great effort has been made by the authors to utilize the feedback that was provided for them to correct. I find it interesting and improved with respect to the original submission. I really encourage the authors to conduct a similar study targeting low- and middle-income countries highlighting the best practices according to their sociodemographic context. However, there are few minor things to adjust related to the introduction section which is lengthy. The authors should consider revising it without putting subheading. | Thank you. It is encouraging that you see the applicability of this work beyond the current context.  We have now removed the subheading from the Introduction. We have also carefully reviewed the manuscript and made further minor modifications.  We agree the Introduction is lengthy, but on this occasion we believe this was necessary given the complexity of place-based approaches, and the broad scope of the review. |
| 3. | Reviewer #1: The manuscript, though long with rather large tables, reasonably addresses the questions set in the objectives. | Thank you. |
| 4. | Reviewer #2: Thank you for considering my suggestions. I think the additional headings to introduce the various aspects of your conceptual model and findings is helpful to readers. | Thank you. |
