# Supplementary material for: Place-based approaches to improve health and development outcomes in young children: A scoping review
Source: PLoS One. 2021 Dec 23;16(12):e0261643. doi: 10.1371/journal.pone.0261643 (PMC8700019; doi:10.1371/journal.pone.0261643)
Supplement: Attachment — Submitted filename: Response to Reviewers November 21.docx [file pone.0261643.s007.docx]

**Place-based approaches to improve health and development outcomes in young children: a scoping review [PONE-D-20-30217]**

**Response to editor and reviewer comments**

| Number | Editor/Reviewer Comments | Response to Editor/Reviewer |
| --- | --- | --- |
| 1. | Journal Requirements:  Please consider including your revision within your response to reviewer comments inside the document of "response to reviewers' comments" to facilitate the understanding of the changes that have been made by the reviewers. In other words, any revision or added references should be included in the document "response to reviewers' comments" and not only as track changes | All changes made to the manuscript are outlined in the comments below, with line numbers provided as reference |
| 2. | Please review your reference list to ensure that it is complete and correct. If you have cited papers that have been retracted, please include the rationale for doing so in the manuscript text, or remove these references and replace them with relevant current references. Any changes to the reference list should be mentioned in the rebuttal letter that accompanies your revised manuscript. If you need to cite a retracted article, indicate the article’s retracted status in the References list and also include a citation and full reference for the retraction notice. | We have made no changes to the reference list in this submission |
| 3. | Reviewer #1: The authors have addressed the issues raised in earlier reviews satisfactorily. The data is clearly presented and description of the tables is easy to follow | Thank you. |
| 4. | Reviewer #2: I do not need to see or comment on this review again, but have a final few comments--the authors can take or leave. Congratulations on completing this! | Thank you. Your feedback on the manuscript has been greatly appreciated. |
| 5. | Abstract Changes suggested here, if accepted, should be made throughout the manuscript. | Changes have been accepted. Please see below. |
| 6. | " Methodologies and methods for evaluating such place-based initiatives were assess" --I am not sure how methodologies and methods are being distinguished here. Suggest clarifying here and anywhere else these two terms are used. BY methodology do you mean study design? | We have clarified that we were referring to ‘study designs and methods’ and changed the wording in the Abstract (line 26), and throughout the manuscript (lines 96-97, 120, 218-219, 387, 650) |
| 7. | You may want to change the word "records" to "reports" ; e.g. 32 reports relating to 12 initiatives. | We have amended the manuscript to use the work ‘reports’ instead of ‘records’ in the Abstract (line 28) and in the Methods section (lines 270 & 276-277) |
| 8. | "Many initiatives were affected by external factors such as policy and funding changes, with unknown impact" This is a finding and if the statement stays in the abstract, it should be repositioned--making it the second last sentence seems the right place. Having said this, I'd say the statement requires clarification. I'm left asking: did these factors mean outcomes couldn't be measured in the studies? Even when I reword the sentence: "External factors such as policy and funding changes had an unknown impact on...." I wonder what I'm meant to understand. | We have repositioned this finding in the Abstract to the second last sentence at your suggestion, and added “within unknown impact *on their effectiveness*” (lines 35-36) |
| 9. | Use of the word "significant". I'd be inclined to remove this adjective. You provide the stats so let them speak for themselves--especially when there is no a priori standard of what is or is not significant change. | We have removed the word ‘significant’ from the Abstract (lines 32, 33, 35) and the main manuscript (lines 575, 579, 608, 610, 631, 739) |
| 10. | Line 33: I would state the number of studies with comparison groups; you mention 6 studies reported more than 1 post baseline measure; how many studies reported only 1 post baseline measure? did you only include data when there was 1+ post measure? | We have now stated the number of studies with comparison groups (line 31).  We are unclear about your second statement. We report findings where studies that report outcomes more than *once* post baseline. We believe our summary in the Abstract is consistent with that reported in the main manuscript. |
| 11. | Line 101: remove conceived; I think all you can say is that some studies describe the intervention as place-based, while others discuss "places" in terms of neighbourhoods, geography or location. | We have removed the word ‘conceived’ (line 101) |
