# Supplementary material for: Place-based approaches to improve health and development outcomes in young children: A scoping review
Source: PLoS One. 2021 Dec 23;16(12):e0261643. doi: 10.1371/journal.pone.0261643 (PMC8700019; doi:10.1371/journal.pone.0261643)
Supplement: Attachment — Submitted filename: Response to Reviewers 16 November 21.docx [file pone.0261643.s008.docx]

**Place-based approaches to improve health and development outcomes in young children: a scoping review [PONE-D-20-30217]**

**Response to editor and reviewer comments 16 November 2021**

| Number | Editor/Reviewer Comments | Response to Editor/Reviewer |
| --- | --- | --- |
|  | Editor Comments |  |
| 1. | Unfortunately, it seems that there is a problem all through the manuscript concerning the focus of the study in term of the measured outcome of health and development of the children. This should be clear in the abstract and introduction not only in the methodology section  As you know health and development outcome is a very broad aspect and it was expected from the authors to clearly indicate in their abstract what aspects of health and development outcome the study focused. | Thank you. We understand that review papers typically specify an outcome of interest, however this was not possible for our Scoping Review.  Place-based initiatives by definition aim to improve multiple and interrelated outcomes across pregnancy and birth, child, parent, family and community domains. Part of the purpose of this review was to understand what outcomes were reported in published studies of place-based initiatives. Therefore rather than approaching this Scoping Review with a pre-determined set of outcomes, we examined the initiatives to identify what outcomes were measured and then we collated and coded them as per the domains and categories presented in Table 1. Different place-based initiatives measured different outcomes, and given this heterogeneity, one of our recommendations (see lines 760 – 769 of manuscript with tracked changes) is that child-focused place-based initiatives use a core set of indicators such as those established by the United Nations Sustainable Development Goals.  We outline our process for examining, collating and coding outcomes in the section ‘Types of outcome measures and other data items of interest’ starting on line 208 of the manuscript with tracked changes. We have now included a summary of this process in our Abstract as follows: (lines 31-34)  “*Place-based initiatives by definition aim to improve multiple and interrelated outcomes. We examined initiatives to determine what outcomes were measured and coded them within the five domains of pregnancy and birth, child, parent, family and community.*” |
| 2. | Please consider to be focused and concise in relation to the following: Aim (This scoping review examines the strength of evidence for the effectiveness of public policy-led place-based initiatives designed to improve outcomes for disadvantaged children, their families and the communities in which they live (be specific: which outcome?) | We agree that our aim is quite wordy, but we need to specify the initiatives that we include in the review, and there are multiple outcomes as described in our response to Comment 1 above. |
| 3. | Methodology: The authors mentioned”. Eleven initiatives used a quasi-experimental evaluation to assess impact, although there were considerable design variations within this…..” (impact on what….). The authors have to mention in their design the aspects of the impact they have targeted in their study. | Our eligibility criteria were that an impact evaluation study had been conducted, and that at least one child outcome had been reported (lines 164 -167). We wanted to describe the full range of outcomes reported in the included studies, so we did not pre-specify particular outcomes. Please also see our response to Comment 1 above. |
| 4. | The results: The authors mentioned” Across the 83 outcome domains reported in the 11 studies with a comparison group, 30 (36.4%) demonstrated a positive outcome, and all but one initiative demonstrated a positive outcome in at least one outcome measure. Of the six studies that examined outcomes more than once post baseline, 10 from 38 outcomes (26.3%) demonstrated positive sustained results. (Again, what were theses outcomes?) | After we collated and coded all the outcomes reported in the included initiatives, we described 30 outcome categories across the 5 domains of pregnancy and birth, child, parent, family and community. These are provided in Table 1. It is not feasible to list all 30 in the Abstract. |
| 5. | Introduction: line 49, 51,52 again, please specify which outcome (for the health aspects as well as the developmental domains) What were the study looking for are not clear | We have now provided some examples of the types of outcomes typically addressed by place-based initiatives in each domain (lines 51-53). As outlined in response to Comment 1 above, we were not looking for a pre-determined outcome or set of outcomes.  The text we have inserted is in italics below:  Such initiatives address not just child outcomes *(e.g. academic, social-emotional, physical, cognitive)*, but also the parent *(e.g. physical/mental health, education, employment)* , family *(e.g. home learning environment, parenting style)* and community *(e.g. cohesion, safety, services)* circumstances that impact on child trajectories [5]. |
| 6. | Line 61 what do you mean by (7, p21)! You should not mention the page number. | Adding a page number for a direct quote enables readers to find the source easily and makes it clear that these are not our own words. Our understanding of the Vancouver reference style is that the page number should be included when directly quoting the exact wording of a source <https://guides.library.uwa.edu.au/c.php?g=324981&p=2177205>  Nevertheless we have noticed that there should be no comma between the reference number and the page number so we have now removed this.  Please advise if *PLOS One* varies from standard Vancouver referencing style in this regard and we will remove the page number. |
| 7. | Again line 64: which outcome (s) the study addressed? | We have added the word ‘multiple’ to this line (now line 67 in the manuscript with tracked changes). Please also see our response to Comment 1 above. |
| 8. | Line 91, 92: The authors mentioned “There is no contemporary literature review that examines evidence of the effectiveness of place-based initiatives for children in their early years” be specific again …. Initiatives for which outcome, | Please see our response to Comment 1 above. |
| 9. | Line 114 to 115 : The authors mentioned “This review focuses on early childhood initiatives that target (but are not necessarily limited to) pregnancy to four years”. Again be specific. | We were interested in describing place-based initiatives that target children and their families from (but not necessarily limited to) pregnancy to four years. This criteria is referring to the timing of childhood not outcomes per se. |
| 10. | Methodology: There is a lot of recent interventions studies in middle east region targeted child outcome in general and development the majority of which were community based or facility based and in rural communities,…… that fit and the authors did not mention any. Please consider adding these studies to enrich your methodology and your review. | Thank you for this suggestion. This Scoping Review is part of a larger PhD project focused on Australia’s largest place-based initiative, Communities for Children. Therefore we chose the inclusion criteria of high income countries (line 176) as it is similar to the setting of our study.  We were very encouraged by your previous suggestion (18 October 2021) to conduct a similar study in low- and middle- income countries and are interested in examining place-based initiatives in other settings. We will consider this suggestion once our current study is completed. |
| 11. | The authors considered exactly the exclusion criteria but did not mention in the inclusion criteria what are the combined outcomes they focused on (please consider this) | Thank you. For clarity we have now included a line in the Inclusion criteria that states:  “*Outcomes: goal of improving multiple outcomes for children and their families*” (line 181) |
